# Evolution of Type 2 Vaccine Derived Poliovirus Lineages. Evidence for Codon-Specific Positive Selection at Three Distinct Locations on Capsid Wall

**DOI:** 10.1371/journal.pone.0066836

**Published:** 2013-06-28

**Authors:** Tapani Hovi, Carita Savolainen-Kopra, Teemu Smura, Soile Blomqvist, Haider Al-Hello, Merja Roivainen

**Affiliations:** Virology Unit, Department of Infectious Disease Surveillance and Control, National Institute for Health and Welfare (THL), Helsinki, Finland; Duke-NUS Graduate Medical School, Singapore

## Abstract

Partial sequences of 110 type 2 poliovirus strains isolated from sewage in Slovakia in 2003–2005, and most probably originating from a single dose of oral poliovirus vaccine, were subjected to a detailed genetic analysis. Evolutionary patterns of these vaccine derived poliovirus strains (SVK-aVDPV2) were compared to those of type 1 and type 3 wild poliovirus (WPV) lineages considered to have a single seed strain origin, respectively. The 102 unique SVK-aVDPV VP1 sequences were monophyletic differing from that of the most likely parental poliovirus type 2/Sabin (PV2 Sabin) by 12.5–15.6%. Judging from this difference and from the rate of accumulation of synonymous transversions during the 22 month observation period, the relevant oral poliovirus vaccine dose had been administered to an unknown recipient more than 12 years earlier. The patterns of nucleotide substitution during the observation period differed from those found in the studied lineages of WPV1 or 3, including a lower transition/transversion (Ts/Tv) bias and strikingly lower Ts/Tv rate ratios at the 2^nd^ codon position for both purines and pyrimidines. A relatively low preference of transitions at the 2^nd^ codon position was also found in the large set of VP1 sequences of Nigerian circulating (c)VDPV2, as well as in the smaller sets from the Hispaniola cVDPV1 and Egypt cVDPV2 outbreaks, and among aVDPV1and aVDPV2 strains recently isolated from sewage in Finland. Codon-wise analysis of synonymous versus non-synonymous substitution rates in the VP1 sequences suggested that in five codons, those coding for amino acids at sites 24, 144, 147, 221 and 222, there may have been positive selection during the observation period. We conclude that pattern of poliovirus VP1 evolution in prolonged infection may differ from that found in WPV epidemics. Further studies on sufficiently large independent datasets are needed to confirm this suggestion and to reveal its potential significance.

## Introduction

Polioviruses (Species *Human enterovirus C*, genus *Enterovirus*, family *Picornaviridae*) are considered to be among the most rapidly evolving viruses with an estimated overall nucleotide substitution rate of about 0.01 substitutions per site per year in the capsid coding region [1], [2], [3], [4], [5], [6], [7], [8], [9], [10]. Evolution of polioviruses (PV), like positive strand RNA viruses in general, is based on a combination of polymerase-error –induced point mutations and recombination, followed by enrichment of variants with increased fitness and/or random sampling due to bottleneck transmission [11], [12], [13]. Distinct genetic lineages are rapidly generated already during an acute PV infection of an individual host [14], and divergent lineages have been described during prolonged replication in immune deficient hosts [7], [15]. In the pre-vaccine era, cumulative poliovirus diversification had resulted, within each serotype, in several co-circulating wild-type poliovirus clades, the designated genotypes [16]. While both deletions and recombinations may occur in the capsid protein coding part of poliovirus genome [17], [18], [19], [20] these changes rather rarely persist in WPV lineages, as compared to recombinations occurring in the untranslated and non-structural protein coding parts of the genome. Hence, single nucleotide substitutions accumulating in the capsid coding part are considered to be derived from a linear continuum of successive virus generations, and therefore, suitable for the analysis of substitution rates and patterns of evolution. While the overall accumulation of mutations in the capsid coding region of replicating polioviruses is considered to follow first order molecular clock kinetics, it is known that in the longer run, saturation of nucleotide substitutions will become evident. As regards the timing of onset of the saturation, different types of substitutions, i.e. synonymous vs. non-synonymous or transitions vs. transversions, differ remarkably [6].

Polioviruses, including the live attenuated Sabin strains used in the oral poliovirus vaccine (OPV), usually cause an acute gastrointestinal tract infection lasting for a few weeks, maximally a couple of months. It has been known for decades, however, that the OPV strains are capable of instituting prolonged infection in the tissues of persons with a deficiency in the humoral immune systems [21]. During the prolonged infection, the vaccine virus is known to drift both genetically and antigenically and not infrequently, to cause a paralytic disease in the host – sometimes several years after the administration of the vaccine [22]. The drifted poliovirus strains shed by the persistently infected immune deficient individuals are referred to as immune deficiency associated vaccine derived polioviruses or iVDPV, with the prefix i to distinguish the strains from the circulating vaccine derived polioviruses (cVDPV), which have caused small or moderate sized outbreaks of paralytic poliomyelitis in several countries [23] (www.polioeradication.org; last visited 15 May, 2013). A relatively large cVDPV outbreak with several years duration in Nigeria was recently published, describing multiple independent emergences ofVDPV from the parental Sabin 2 virus [24]. OPV-strain derived polioviruses with genetic features resembling those of the iVDPV strains have also been isolated from environmental specimens [25], [26], [27], [28]. Persons shedding these vastly drifted aVDPVs (a for ambiguous) have remained unidentified. It is not clear if the evolutionary trends occurring in prolonged poliovirus infection are similar to those established for WPV transmission in human populations.

Slovak Republic, a small Central-European country with decades-long record of elimination of wild PV transmission and no reported wild-type paralytic poliomyelitis since 1960, experienced in 2003–2005 an episode of repeated isolations of environmental VDPV strains (SVK-aVDPV2) [27]. The intensive search to identify the aVDPV shedding person(s) provided us with a large number of aVDPV containing sewage samples, and more than 100 SVK-aVDPV2 strains isolated over a period of two years. Both genetic and phenotypic properties of the representative strains resembled those of iVDPV strains described previously. Phylogenetic analysis of 110 complete capsid protein VP1 sequences indicated that all strains were monophyletic and were probably derived from a single dose of the oral vaccine strain Sabin 2 (PV2 Sabin) [29]. In this article we report thorough genetic analysis of these VP1 sequences. Specifically, we describe uncommon substitution patterns, signs of positive selection and inter-lineage recombination during the evolution of the SVK-aVDPV2 strains. In addition, different approaches to determine the time elapsed since the relevant OPV administration were applied.

## Materials and Methods

### Virus strains and generation of sequence data

Poliovirus strains characterized in this study originated from environmental samples. Poliovirus strains with aberrant ITD results were subjected to partial genomic sequencing, and VDPV strains were identified as described before [18], [29]. Complete VP1 encoding region was sequenced from all 110 individual SVK-aVDPV2 isolates (excluding some of the very last ones), often several of them originating from a given sewage sample. The dates of isolation were not evenly distributed through the 22 months monitoring period for the set of 102 unique sequences analysed, for reasons described in the adjacent paper [29]. Partial 3D coding region was sequenced from a portion of the isolates. The GenBank accession numbers of the analysed SVK-aVDPV2 sequences are JX913541-JX913646 for VP1 and JX913647-JX913690 for the partial 3D sequences. For reference, we used the sequence of PV2-Sabin; AY184220). In the absence of relevant sets of WPV2 sequences, the nucleotide substitution patterns in the VP1 sequences of SVK-aVDPVa were primarily compared to those published by Jorba *et al.* [6], GenBank accession numbers EF374000-EF374030, and describing transmission of single-seed descendants of WPV1 in Latin America. The entire capsid coding sequences were manually edited to include only the VP1 coding sequences, later referred to as the Andean WPV1 sequences. Secondly, we used VP1 sequences from a WPV3 outbreak [30], which can also be considered to have emerged after importation of a single seed virus to Finland in 1984 (X04468, FJ84160-79). The recent cVDPV2 report from Nigeria including 361 VP1 sequences (JX274980-JX275382) derived from strains of one single emergence of VDPV [24] was finally included in the comparison. In addition, the Supporting evidence section presents supplementary sequence analyses with references to relevant GenBank accession numbers.

### Phylogenetic analysis

Phylogenetic relationships between the SVK-aVDPV2 sequences and those downloaded from the GenBank were analysed with the MEGA software package, versions 4 [31] or 5 [32] as described in the adjacent article. Primarily, the neighbour-joining (NJ)-method with the maximum composite likelihood substitution algorithm and gamma distributed substitution rate variations among sites and heterogeneous evolution rates among lineages was used. In addition, a Bayesian Monte Carlo Markov Chain (MCMC) method was used as implemented in the BEAST version 1.5.1 and 1.7.4 [33]. The analyses were performed using a relaxed molecular clock model (the uncorrelated log-normal distributed model) [34], the general time reversible (GTR) model of substitution with gamma distributed substitution rate variation among sites, and Bayesian skyline demographic model. The Bayesian analyses were run for 100 million states and sampled every 10000 or 100000 states. Posterior probabilities were calculated with a burn-in of 1 million states and checked for convergence using Tracer version 1.4.1 and 1.5. The analyses were carried out on the Bioportal server, University of Oslo (www,bioportal.uio.no; last visited 18 April 2013) [35].

Partial 3D sequences were first manually edited to a standard length (427 nt), aligned with Clustal X and analysed for genetic relationships as above. Potential recombination between different lineages was studied by visual examination of the branching orders and their probabilities in the two NJ trees.

### Substitution patterns, rate of evolution and estimation of date of divergence from PV2 Sabin

The proportions of the four nucleotides and the patterns of nucleotide substitution in entire codons and in the three individual codon positions were calculated for the SVK-aVDPV2 VP1 sequences using the MEGA5 software and compared with the corresponding values of the two previously generated sequence sets [6], [30] For the assessment of substitution rates, MEGA was used to identify the numbers of 2- and 4-fold degenerate sites in the sequence set and calculate the number of transversions in these sites for each strain with regard to the PV2 Sabin sequence. The detection date of a given strain was expressed as days between the date of collection of the respective sewage sample and that yielding the index strain of the episode (#783, 2 April 2003). The Linear Regression method exploiting the least square principle in the Microsoft 2010 Excel program package was used to assess the correlation between the sequence divergence and the date of detection, and to calculate the coefficient *m* in the formula y  =  *m•* x + b, where y  =  number of observed nucleotide differences and x  =  days since 2 April 2003, and b  =  value of y when x = 0. For the calculation of the apparent evolutionary rates of different nucleotide categories the corresponding *m* value was multiplied by 365 and the result was divided by number of sites in the analysis to get number of substitutions per site per year. The formula was also used to estimate years (before 2 April 2003) since the divergence from PV2 Sabin by substituting 0 for y, and solving x in the equation followed by division of the result by 365. In addition, for the estimation of the rate of evolution and divergence times for the VP1 sequences a Bayesian Monte Carlo Markov Chain (MCMC) method was used as implemented in the BEAST version 1.7.4 [33]. The most probable sequence of the most recent common ancestor (MRCA) of SVK-aVDPV strains was estimated using maximum likelihood method implemented in MEGA 5.0 software. The GTR model of substitution with gamma distributed substitution rate variation among sites was used in this analysis.

### Distribution of amino acid substitutions

MEGA5 was used to identify variable sites and the non-singleton variable sites in amino acid sequences deduced from the VP1 coding nucleotide sequences of the 102 unique SVK-aVDPV2 strains. Distribution of these sites through the sequence was visualized using the Excel program. The locations of variable amino acid sites in virion structure were examined using Jmol [36][] and the 3-dimensional structure model of poliovirus pentamer based on x-ray crystallographic analysis of type 2 poliovirus strain Lansing (PDB ID: 1IEAH) [37].

Signs of selection at codon level were sought for by determining the codon specific differences in synonymic and non-.synonymic substitution rates using the HyPhy program [38] in the MEGA5 package, and the single likelihood ancestor counting (SLAC), fixed effects likelihood (FEL), and internal fixed effecs likelihood (IFEL) methods [39] in the Datamonkey web service (http://www.datamonkey.org/; last visited 30 Oct 2012) [40].

## Results

### Common path of evolution and designated lineages of Slovakian VDPV strains

As reported in the adjacent article [29], the 110 environmental type 2 poliovirus strains isolated in Slovakia in 2003–2005 were monophyletic and showed closest relationship to the PV2 Sabin strain in all genomic regions, although widely divergent in the capsid protein coding sequences, and thus designated as VDPVs. Since the number of PV2 VP1 sequences in the GenBank is much larger than that of complete genomes, we made an additional phylogenetic analysis with PV2 VP1 sequences available in the GenBank on April 15, 2013. The results shown in Fig. S1 again indicate that the Slovakian environmental strains are monophyletic and have no known relatives significantly closer than PV2 Sabin; we thus refer to them in this paper as SVK-aVDPV2 strains.

To study the assumed initially common evolutionary pathway of the SVK-aVDPV2 strains we searched for conserved nucleotide substitutions compared to the sequence of PV2 Sabin. At 22 out of 903 nt sites a fixation of a mutation had occurred before divergence of the current VDPV lineages, i.e., the nucleotide was fully conserved among the VDPV strains but different from that of PV2 Sabin. At 11 other sites, all the VDPV strains were also different from PV2 Sabin but a few strains were different from the majority of the VDPV strains. At further 40 sites, majority of the VDPV strains showed a conserved substitution while 1–5 strains, isolated relatively late during the episode, had the same nucleotide as PV2 Sabin, possibly representing back mutations (Table 1). At one site, the apparent back mutation was seen in a subcluster of six strains and a single related strain. Ten out of the 33 sites of the first two categories were transversions while only three of the 40 sites showing back mutations were transversions. Eleven of the listed 73 mutations were associated with an amino acid substitution as shown in Table 1.

**Table 1:** VP1 gene point mutations that have occurred in the PV Sabin -derived Slovakian aVDPV2 strains before the study period.

Nucleotide substitution			Amino acid substitution		
Site	Sabin 2	SVK VDPV	Site	Sabin 2	SVK VDPV
**27**	**C**	**A**			
33	A	G			
45	A	G			
**48**	**T**	**G/a**	16	N	K
51	A	G			
56	T	C/t	19	V	A/T
59	C	T	20	P	L
**72**	**C**	**A**			
81	G	A			
**108**	**C**	**A**			
135	A	G/t			
**147**	**A**	**C/t**			
181	G	A	61	V	I
183	G	A/g			
207	A	G			
249	A	G			
304	A	G/t	102	S	A
**305**	**G**	**C**/g	102	S	G/C
308	G	A/g	103	R	K/R/Q
318	G	A/c/t			
327	A	G			
342	T	C			
352	C	T			
**378**	**A**	**T/c**			
428	T	C/t	143	I	T/P/I
459	A	G			
507	A	G			
511	A	G/a	171	N	D/N
519	T	C			
**531**	**G**	**T**/c/**a**			
567	G	A			
**568**	**C**	**A/c**	190	P	T/P
**600**	**A**	**T/c**			
612	G	A			
**661**	**G**	**T/c**	221	A	S/P
668	**C**	**G/c/a**	223	T	S/T/R/N
816	A	G			
**839**	**T**	**A**	280	F	Y
846	A	G/c			
858	T	C			

Included are 33 nt sites where all SVK-aVDPV2 differed from PV2 Sabin, and seven sites where a few strains showed back mutation. This did not always result in reversion at amino acid level because of other coinciding mutations in the codon. Nucleotide transversions are indicated by bold phase. In addition, synonymous transitions were seen in sites 15, 39, 63, 66, 138, 177, 192, 213, 228, 258, 276, 288, 291, 364, 399, 405, 465, 477, 504, 516, 522, 543, 594, 606, 615, 654, 675, 690, 756, 789, 828, 885, 897.

**Figure 1:**
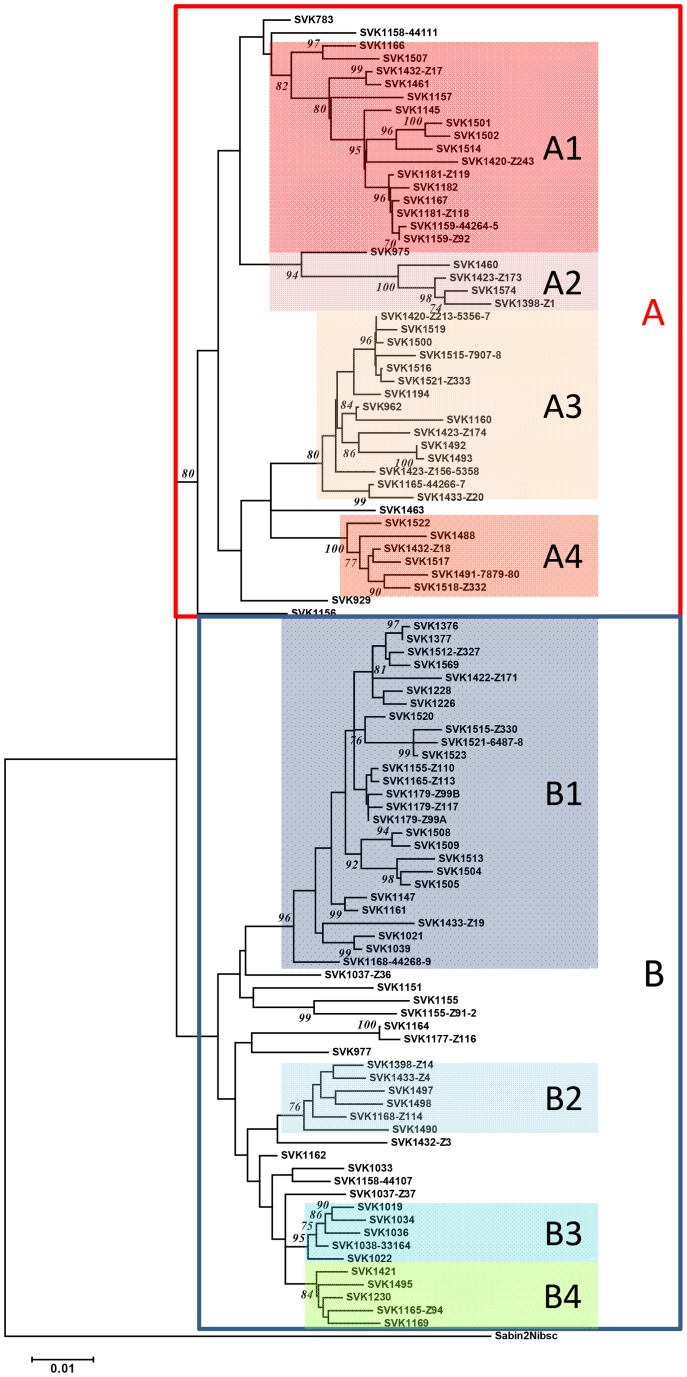
Designated lineages of Slovakian vaccine derived poliovirus (VDPV) strains isolated in 2003–2005. NJ tree based on phylogenetic analysis of 102 unique VP1 sequences from the VDPV strains is shown with type 2 poliovirus strain Sabin as outgroup. Bootstrap values less than 70 are omitted. Strains outside the coloured boxes are referred to as orphan strains in the text.

A NJ tree constructed with the PV2 Sabin alone as an outgroup revealed the same two major clusters and topology of subclusters previously found by also including unrelated type 2 poliovirus sequences in the analysis (Hovi et al. 2013, adjacent), but some of the relevant bootstrap values were lower. For purpose of discussion we designated bootstrap supported sub-clusters with five or more unique strains as lineages A1-A4 and B1-B3; this subgrouping left out several “orphan” strains in both major clusters (Fig. 1). The phylogenetic tree construction with the Bayesian MCMC method showed high posterior probabilities to the designated subclusters but somewhat different topologies for some of the orphan strains (Fig. S2)).

### Nucleotide substitution patterns in VP1 coding sequences

The analysis of nucleotide composition of the VP1 sequences of the studied VDPV strains suggested minor differences as compared to those of the Andean WPV1 strains, e.g. slightly higher proportion of purines but, as judged from the sequences of PV2 Sabin and the few WPV type 2 poliovirus strains used in the adjacent paper [29] (GenBank accession numbers: Lansing AY082680, Lederle II AY082678, MEF-1 AY238473, and W2 AY082679), these minor differences might be just random variation or due to different serotype rather than a difference between VDPV and WPV strains (data not shown).

The number of variable sites within the VP1 gene among the SVK-aVDPV2 strains was only marginally less than that between the SVK-aVDPV2 strains and the assumed ancestral PV2 Sabin strain (Table 2). Substitution patterns were evaluated by determining relative substitution rates and modes of the four nucleotides using the MEGA5 software. Inclusion of the PV2 Sabin strain in the analysis did not change the patterns remarkably from those obtained with the 102 VDPV strains alone. Most of the substitutions between SVK strains were transitions (Table 3). Substitution frequencies were somewhat different in different codon positions, with the 3^rd^ position showing a pattern very similar to that of the overall picture. In the 1st and the 2^nd^ positions the proportions of transversions were somewhat higher. The 2^nd^ codon position showed clearly different transition pattern compared to the 1^st^ and 3^rd^ positions. The rates of pyrimidine transitions (TC/CT) and the rate of G to A were higher in the 2^nd^ codon position in comparison the two other positions. At the 1^st^ position, G appeared to be relatively conserved and at the 2^nd^ position just the opposite, substitution of G to one of the other three nucleotides represented about 46% of all substitutions (Table 3).

**Table 2:** Number of conserved and degenerate sites in VP1 coding region.

Sequence set*	*N*	Conserved (variable)	Non-degenerate	Degenerate	2-fold degenerate	4-fold degenerate
SVK+S2	103	533 (370)	577	326	115	142
SVK	102	553 (350)	577	326	117	142

* SVK, Slovakian aVDPV2 strains; S2, PV2 Sabin strain [48].

N, number of strains in the set.

**Table 3:** Codon position-dependent patterns of nucleotide substitutions among the 102 Slovakian VDPV strains.

Codon position	Original nucleotide	Substitute nucleotide			
		A	T	C	G
Total codon	A	-	*1.72*	*1.94*	**17.47**
	T	*2.07*	-	**25.26**	*1.82*
	C	*2.07*	**22.38**	-	*1.82*
	G	**19.79**	*1.72*	*1.94*	-
Position 1	A	-	*2.42*	*2.15*	**15.06**
	T	*3.15*	-	**23.24**	*3.75*
	C	*3.15*	**26.15**	-	*3.75*
	G	**12.63**	*2.42*	*2.15*	-
Position 2	A	-	*3.41*	*4.63*	**20.4**
	T	*4.04*	-	**7.56**	*2.17*
	C	*4.04*	**5.57**	-	*2.17*
	G	**37.98**	*3.41*	*4.63*	-
Position 3	A	-	*1.35*	*1.5*	**17.91**
	T	*1.52*	-	**26.98**	*1.42*
	C	*1.52*	**24.31**	-	*1.42*
	G	**19.21**	*1.35*	*1.5*	-

Each entry shows the probability of substitution (r) from one base (row) to another base (column) [49]. For simplicity, the sum of r values is made equal to 100. Rates of different transitional substitutions are shown in **bold** and those of transversional substitutions are shown in *italics*. Evolutionary analyses were conducted in MEGA5 [31].

The overall Ts/Tv bias and the Ts/Tv rate ratios for purines and pyrimidines were definitely lower in the SVK-aVDPV2 dataset than those found for the WPV1 Andean and the Nigerian cVDPV2 sequences. The smaller Finnish WPV3 dataset showed figures closer to those of the SVK-aVDPV2 except the rate ratio for purines which was similar to that of the Andean WPV1 sequences. The Nigerian cVDPV2 dataset had coefficients close to those of the Andean WPV1 (Table 4). When this type of substitution pattern analysis was broken down to the three codon positions separately, the differences between SVK-aVDPV2 and the WPV1 and WPV3 datasets were amplified with highest differences at the 2^nd^ codon position, where the WPV sequence sets showed hundred fold higher preference of transitions compared to the VDPV set of sequences. The Nigerian cVDPV2 sequences had coefficients closer to those of the SVK-aVDPV2 (Table 4). While the 1^st^ and 3^rd^ positions showed definite variation in the various Ts/Tv coefficients between the four datasets, no pattern typical of VDPV was observed on the basis of these positions. We also made the Ts/Tv analysis to several smaller sets of aVDPV and cVDPV sequences. The overall coefficients were usually close to those of the Andean WPV1, but at the 2^nd^ codon position both overall Ts/Tv bias values and the rate ratios for the two nucleotide categories of the analysed aVDPV and cVDPV sequence sets resembled those of the the SVK-aVDPV2. The Ts/Tv coefficients and some other parameters of these sequence sets are shown in in the Table S1.

**Table 4:** Codon position-wise analysis of transition preference in nucleotide substitutions during evolution of poliovirus VP1 coding sequences in different contexts.

			Sequence set			
			Slovakian VDPV2	Andean WPV1	Finland WPV3	Nigerian cVDPV
No. of strains			102	31	21	361
Test category		Codon position				
Transition/transversion bias		1,2,3	5.6	10.6	7.3	9.3
		1	3.3	5.9	15.2	12.6
		2	2.2	448.5	233.3	3.7
		3	7.6	12.9	5.9	10.5
Transition/transversion rate ratio	Purines	1,2,3	9.6	19.1	18.3	15.6
		1	4.0	5.9	42.1	12.5
		2	9.4	709.1	1000.0	9.6
		3	12.6	26.3	13.5	17.8
	Pyrimidines	1,2,3	13.0	25.5	10.9	22.0
		1	10.8	22.9	0.0	47.3
		2	1.6	1000.0	220.2	6.3
		3	18.0	25.3	10.6	24.5

### Analysis of potential targets of codon-specific selection

**Figure 2:**
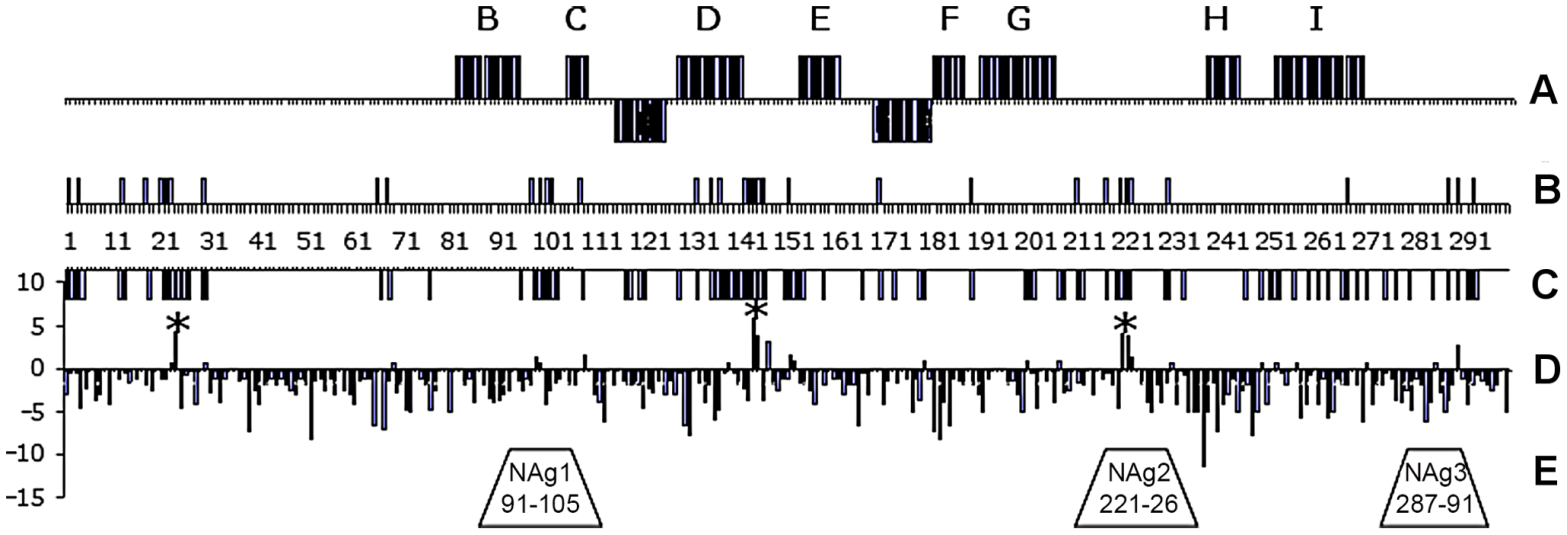
Distribution of selected motifs and individualamino acid sites along the VP1 sequence. Panel A, location of beta strands B to I and alpha helices (under horizontal line) predicted by the MEGA5 software; Panel B, non-singleton variable sites; Short vertical lines and umbers between panels B and C indicate the number of amino acid sites; Panel C, all variable sites, Panel D, Codon-specific selection, normalized dNS-dS, statistical significance (P<0.05) indicated by * for sites positively selected according to HyPhy; Panel E, location of known neutralizing monoclonal antibody inducing antigenic sites.

As many as 93 deduced amino acid sites showed variation between the individual SVK-aVDPV2 strains. The greatest difference, noted at 22 out of 301 amino acid sites, were between strains #1155 (an orphan strain in cluster B) and two strains from the other major cluster, #1574 (cluster A2) and #1488 (cluster A4). Thirty one non-singleton variable sites were distributed throughout the entire sequence but were relatively more frequent in the N-terminal tenth of the protein, and in the known antigenic sites and surface exposed loops of the protein and some in the beta-strand D facing the inner surface of the capsid shell (Fig. 2).

**Figure 3:**
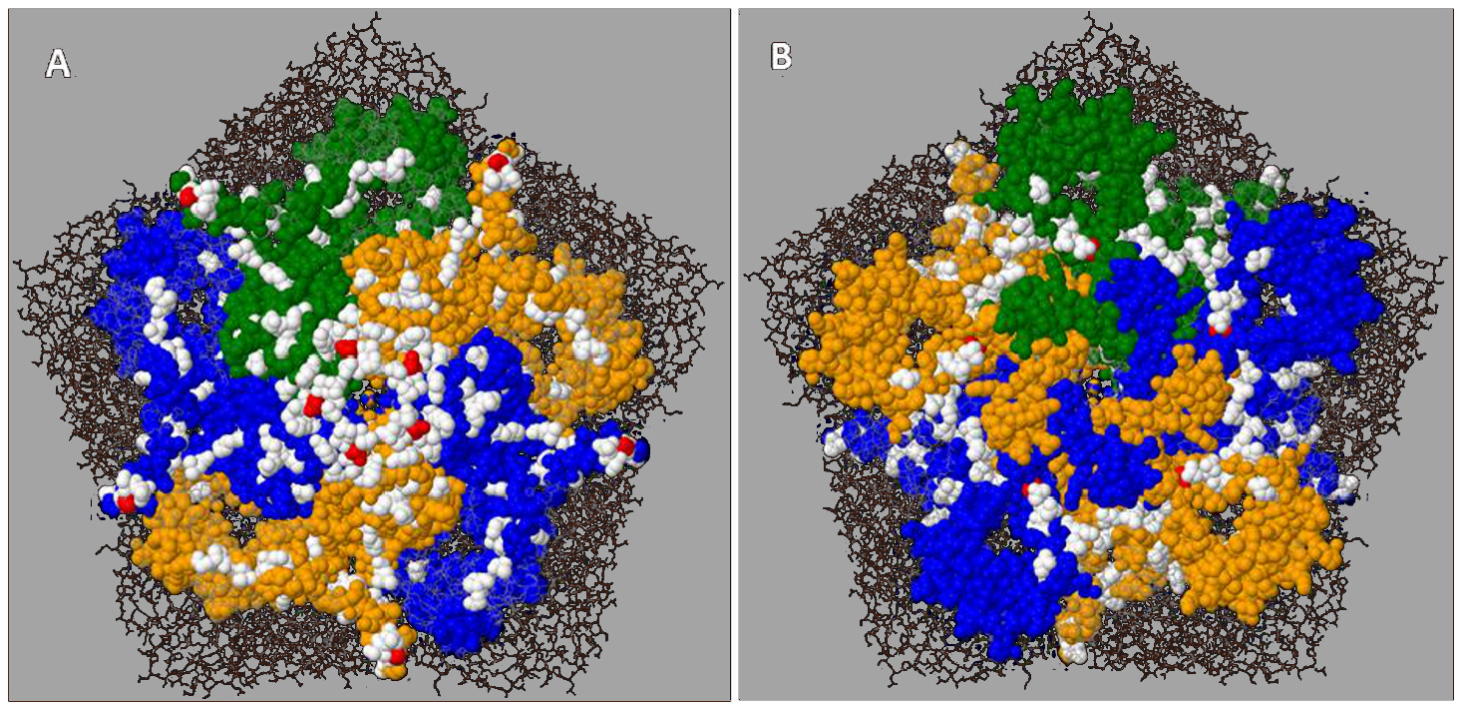
Locations of positively selected codons in 3-dimensional structure model of poliovirus capsid protein pentamer. For clarity, only VP1 is shown in space filling model with different colours in different protomers. Panel A, outside view; panel B, view from inside of capsid shell. Locations of variable amino acid sites are labelled with white colour and those showing evidence for positively selected codons (¤24, ¤144, and ¤221) with red.

Codon usage in the three sets of VP1 sequences was analysed using the MEGA5 software. Differences between the datasets were seen in the usage of codons for some amino acids coded by multiple codons (Table S2) but there was no indication for a pattern typical of the VDPV sequences and different from those of the two WPV sequence sets. The ratios of synonymous (S) versus non-synonymous (NS) substitutions at individual codons were estimated to analyse potential codon-specific selection pressures during the evolution of the SVK-aVDPV2 strains. At most codons the NS/S ratio was very low suggesting negative selection. However, the HyPhy-program in the MEGA5 software package suggested positive selection for three codons, ¤24, ¤144, and ¤221 (Fig. 2; Fig. 3). Applying the Datamonkey web service using different algorithms confirmed the significance of these signals. The SLAC method suggested positive selection in exactly the same three sites, while the FEL and the IFEL methods suggested positive selection, in addition to the three above ones, at sites ¤147 and ¤222, respectively. Only one out of the three regions with codons suggested as targets of positive selection (¤221–222) was located in a known neutralising antibody inducing antigenic site.

**Figure 4:**
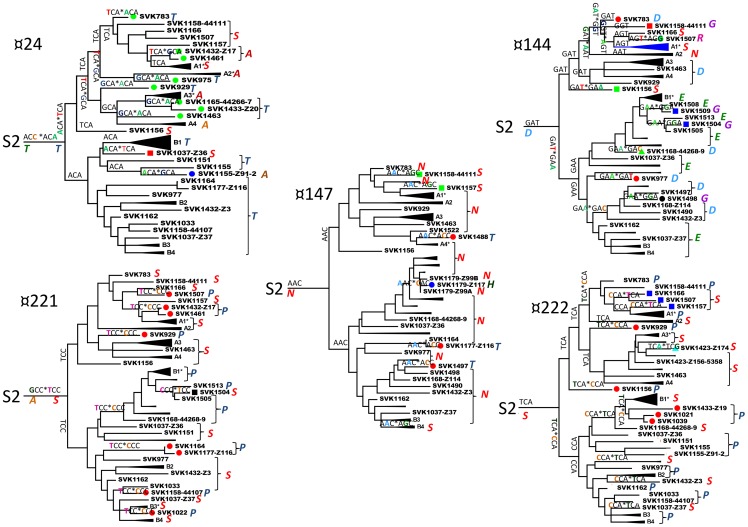
Putative emergence of mutations resulting in observed amino acid substitutions at positively selected codons. Possible historic occurrence of relevant point mutations at sites ¤24; ¤144, ¤147; ¤221, and ¤222 is indicated by tandem nucleotide triplets with a nucleotide change in one codon position. > stands for direction of change. Single triplets between branching points indicate maintenance of unchanged sequence. Nucleotide triplets refer to tree branches below (horizontal lines) or at right hand side (vertical lines). Lineage labels with sign * represent majority of designated lineage members, with one or more individual strains showing different amino acid (or just different codon). Most latter strains are also labelled with coloured symbols to mark separation from lineage. Note that on many occasions there are multiple possible evolutionary pathways of which only a single hypothetical alternative is presented.

Accumulation of different amino acid substitutions in these sites and possible steps of the corresponding point mutations are shown in Fig. 4. In all five sites, many of the amino acid substitutions appeared to have occurred several times independently in diverse lineages and usually, but not exclusively, through identical point mutation. Occasionally, a reverse mutation seemed to have occurred resulting in reappearance of the initial amino acid in a sublineage. At ¤24, threonine was frequently replaced by serine or alanine in cluster A, and occasionally reverted to threonine. In cluster B the original threonine persisted with few exceptions. At ¤144 the original aspartic acid persisted in cluster A in some early isolates and some lineages but the rest of strains showed a range of different amino acids. In cluster B, aspartic acid appeared to be initially replaced by glutamic acid, followed by reversion back to aspartic acid in some strains. At ¤147 the initial asparagine persisted in all designated lineages except B4 but was replaced in several strains either by serine or by threonine, and once by histidine. At ¤221 the initial alanine appeared to be rapidly changed to serine, which subsequently was replaced by proline in several lineages independently, and once back to serine (#1504). At ¤222 the initial serine mostly persisted in cluster A, but, in cluster B, appeared to be replaced by proline. Both major clusters contained, however, individual strains and sublineages showing the alternative amino acid, i.e. proline in cluster A and serine in cluster B (Fig. 4).

The sequences coding for VP1 of the Andean type 1 WPV showed no significant signs of positive selection in any of the codons, as also originally reported for the entire P1 sequences [6]. The Nigerian cVDPV2 dataset was also analysed with the HyPhy program and no evidence for positive selection was obtained. Likewise, the 21 VP1 sequences collected during the WPV3 outbreak in Finland in 1984 [30], showed no statistically significant signs of codon-specific selection in corresponding analysis, although some positive values for the dNS-dS were recorded. To test the influence of the number of sequences in the analysis, we reanalysed the SVK-aVDPV2 sequences separately for clusters A and B. The selected codons still gave a positive dNS-dS signal but the statistical support for selection was lost (not shown).

### Evolution rates of SVK-aVDPV2 strains and computed time of divergence from PV2 Sabin

**Figure 5:**
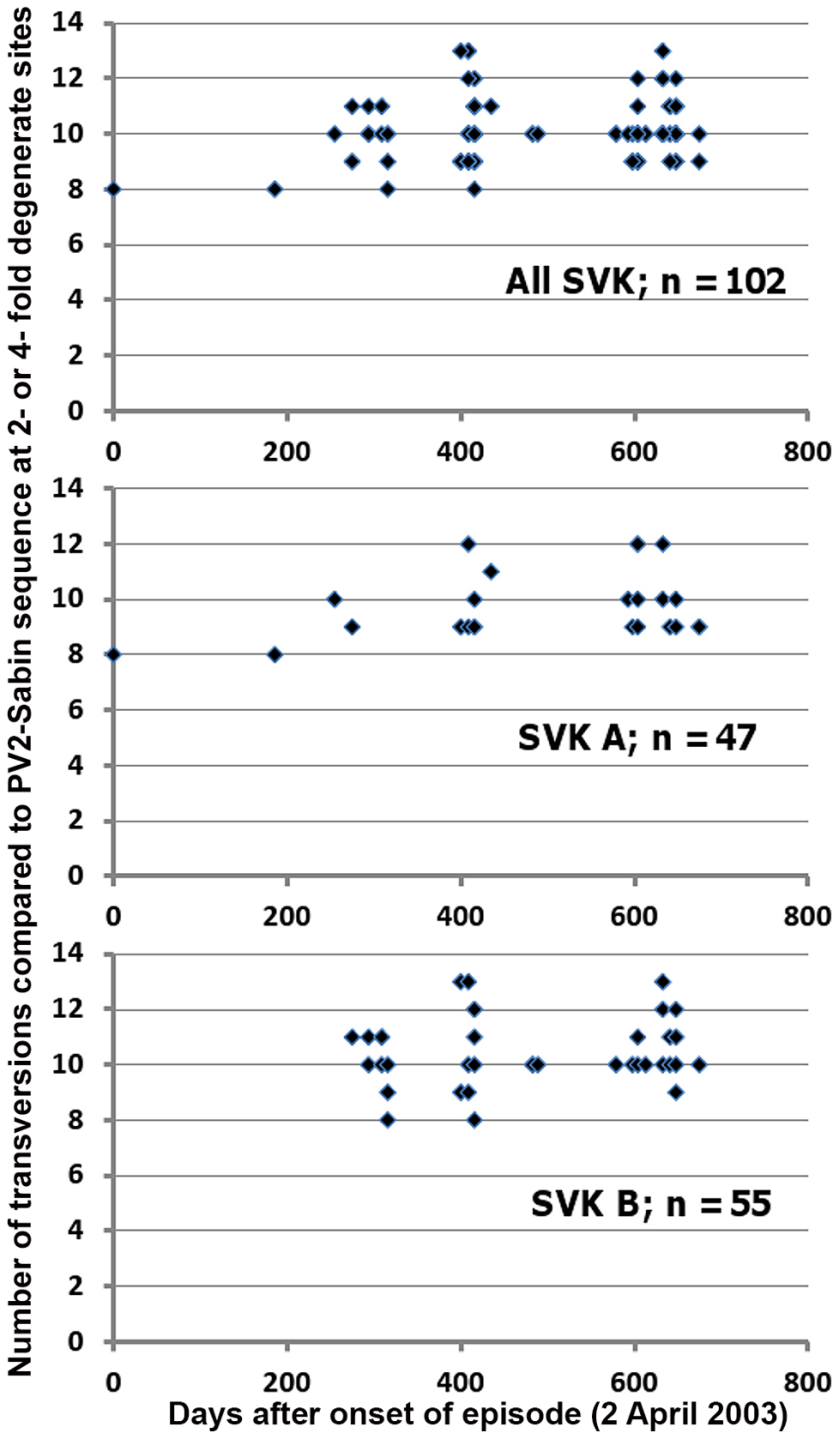
Estimation of transversion accumulation rate in degenerate sites of VP1 coding region of Slovakian aVDPV2 strains. Calculations are based on substitutions occurring during the 22 months monitoring window in 2003–2005. Time in x-axis is given in days since onset of the episode, 2 April 2003. Y-axis indicates number of transversions in 2-fold and 4-fold degenerate sites compared to PV2 Sabin sequence. Multiple identical values at any two-dimensional point were omitted from calculation of linear regression coefficients (See Table 5). Entire sequence set (uppermost panel) and major clusters A and B were used as starting materials.

The analysed SVK-aVDPV2 VP1 sequences differed from that of PV2 Sabin by 12.5–15.6%. Assuming linear accumulation of substitution at a rate of 1% per year [3] this would mean origin from a vaccine dose administered at least 12 years before the onset of this episode. As the rate of transitions representing most of the accumulating substitutions is known to slow down because of saturation of substitutions [6] only transversions in the 2- or 4-fold degenerate sites were considered in further linear regression analysis. While the calculated evolution rate appeared reasonable, 0.0013 substitutions per site per year, the predicted divergence time from PV2 Sabin, assuming linear accumulation of transversions in these sites, was about 30 years (Fig. 5, Table 5). For comparison, if all substitutions at all sites were included in the analysis, the value of the coefficient *m* was negative (not shown). For several time points there were multiple strains with unique sequence but identical difference values. When identical replicate values were excluded from the analysis the subsequent apparent evolution rate was almost doubled and the prediction of years since divergence from PV2 Sabin yielded 14.4 years (Table 5, Fig. 5). One further approach applied was based on reduction in the number of representatives from different sublineages in the analysis. A set of 21 strains was selected covering both all designated subclusters and a few orphan strains. The apparent rate of accumulation of transversions in the two-fold or four-fold degenerate sites and the predicted time of divergence from PV2 Sabin were between the two values given above for the entire set of 102 strains (Table 5). We also made an attempt to see if the rates of substitution would be different in different lineages, using the duplicate reduction principle described above. The results indeed showed a considerable range of apparent evolution rates (Table 5). The Bayesian relaxed molecular clock model was also used to analyse the evolution of the SVK-aVDPV strains. It suggested a mean evolutionary rate of 0.0213 (high-probability distribution [93% HPD] range 1.79–2.49×10^−2^) with all strains included, and a predicted divergence from the MRCA of clusters A and B of 2.8 (2.3–3.43) years (Fig. S2). If constant substitution rates of 1.1×10^−2^ [6], [24] to 2.1×10^−2^ were assumed, the divergence between the estimated MRCA and PV2 Sabin equalled to 6 to 18 years of evolution.

**Table 5:** Evolution rates since 3 April 2003 based on number of Tv substitutions at 2- or 4-fold degenerate sites (N = 257) and estimated time since PV2 Sabin if linear rate.

Sequence set	N	Dates*	m∧	Evolution rate#	b value¤	Time in years
All SVK-aVDPV2	102	0–675	0.000914	0.0013	9.60	28.8
SVK unique points@ only	51	0–675	0.001775	0.0025	9.34	14.4
Cluster A	47	0–675	0.002321	0.0033	8.60	10.2
Subcluster A1	16	400–648	0.000386	0.0006	9.09	64.5
Subcluster A3	15	255–648	**−**0.0022	NR	NR	NR
Cluster B	55	275–675	0.001133	0.0016	9.87	23.9
Subcluster B1	27	294–675	0.003133	0.0045	8.36	7.3
Subcluster B2	6	416–633	0.00728	0.0103	6.59	2.5
Selected sample&	21	0–648	0.001384	0.0018	9.72	

* Days since collecting sample yielding #783.

∧Linear regression coefficient.

# Number of nt changes/site/year.

¤ y – axis cutting point.

@For any time point with more than one identical p-difference, the value included in calculation only once.

& Representatives of of all lineages and some orphan strains included.

NR, not relevan.

### Partial 3D-sequences and signs of recombination

**Figure 6:**
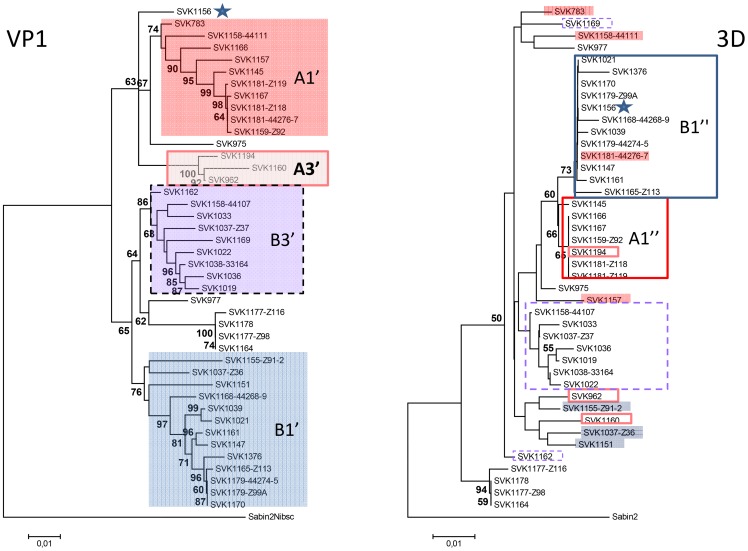
Incongruent branching order of partial Slovakian aVDPV2 3D sequences as compared with corresponding VP1 sequences. Designated lineages in VP1 (Panel A) are labelled with ‘ as they differ somewhat from those in Fig. 1. Strains with incongruent topology in the 3D tree (Panel B) are shown in variable colours.

Partial 3D sequences of variable length were obtained from 44 SVK-aVDPV2 strains and edited manually to equal length, 427 nt corresponding to positions 6740–7166 in the #783 sequence. Sequences from virus strains lacking the corresponding VP1 sequence were removed resulting in 42 sequences which were edited at both ends to obtain a 423 nt sequence in frame (6742–7164) and capable of coding 141 amino acids. When aligned with the corresponding sequence of PV2 Sabin, 70 sites were variable including 12 fixed mutations. None of the latter ones were involved in amino acid substitutions, which were very rare in this genome region – there were only 3 variable sites out of 141. The p-difference between PV2 Sabin and the 42 SVK-aVDPV2 strains ranged from 5.9 to 8.7 per cent. Fifty seven sites were variable among the VDPV strains, and the maximum p-difference was 4.3 per cent. All analysed SVK-aVDPV2 3D sequences were monophyletic and apparently descended from PV2 Sabin (Fig. 6). The analysed subset of VDPV strains was biased, as compared to the entire VP1 sequence set analysed above, by containing relatively more of the designated cluster B sequences, and by lacking strains isolated late in 2004 or in 2005. Yet, the clustering of the corresponding VP1 sequences largely resembled that obtained with the full set of VP1 sequences; only the designated lineages A4 and B2 that contained mainly late isolates were missing. Interestingly some of the orphan-labelled strains now clearly clustered with the neighbouring lineages (Fig. 6). This subset of VP1 sequences had a maximum mutual p-difference of 8 per cent and a range of p-differences from 12.7 to 14.8 per cent compared to PV2 Sabin. The different Ts/Tv coefficients were similar to those of the entire VP1 sequence set (data not shown). Phylogenetic analysis of the partial 3D sequences using the NJ algorithm revealed a tree topology clearly different from that obtained with the corresponding VP1 sequences. Strains from several of the designated lineages in the VP1 tree showed incongruent segregation in the 3D tree. For instance, four out of ten strains of the VP1 lineage A1′ were definitely outside the moderately supported A1′′ cluster in the 3D tree, one of them within cluster B1”. The 3D cluster A1′′ also included a strain from the VP1 lineage A3′, whose other two members appeared to cluster with a few members of VP1 lineage B1′ but with low bootstrap support. Another loose group without bootstrap support (topmost in Fig. 6) was shared by two members of the VP1 A1′ lineage and two strains from the other major cluster B.

## Discussion

In this paper we have described a detailed analysis of substitution patterns and trends of evolution among 102 type 2 VDPV strains isolated from environmental specimens during a 22 month period in Slovakia. The analysis revealed differences compared with those found in previously reported wild type 1 poliovirus evolution. The observed differences included lowered preference of transitions over transversions, especially in the 2^nd^ codon position, and evidence of positive selection in a few codons coding for surface exposed amino acids in the VP1 protein. Topologies of VP1 and partial 3D sequences in the NJ trees showed numerous incongruences suggesting frequent inter-lineage recombinations during the evolution of the SVK-aVDPV2 strains.

### Patterns of nucleotide substitution

Since our primary interest was to analyse evolution of the SVK-aVDPV2 during the 22 months follow-up window, we excluded the assumed parental PV2 Sabin strain from these analyses. Initial adaptation of PV2 Sabin to human tissues is known to result in certain type of substitutions rapidly after administration of the attenuated poliovirus vaccine. These markers of early adaptation, such as substitution of amino acid 143 of VP1 were also seen in the SVK-aVDPV2 strains but excluded from the evolutionary analysis. Likewise, the other aVDPV and cVDPV sequence sets were analysed without the respective parental Sabin strains.

While no VDPV specific patterns could be identified for nucleotide composition or codon usage, the patterns of nucleotide substitution were definitely different from those seen in the studied WPV1 sequence set. Preference of transitions over transversion, expressed as overall transition-transversion bias or the Ts/Tv rate ratios for both families of nucleotides, was strikingly lower in the SVK-aVDPV2 sequence set than in the sequence sets derived from the Andean WPV1 lineage transmission. This difference was dramatically increased if the analysis was based on the codon positions 2 only. Here, the rate of transversions was extremely low in the studied WPV1 sequence set. Unfortunately, similar sequence sets are not available for the wild type 2 poliovirus as WPV2 was eradicated already before the year 2000. We therefore included a small set of WPV3 sequences in the analysis. These WPV3 strains can be considered to be derived from a single seed strain imported to Finland in 1984 [14]. The mean p-distance between these sequences was, however, relatively small and the strains had been collected within a few months period. The Ts/Tv coefficients for all the codon positions of the WPV3 sequence set were variable but those for the 2^nd^ position were similar to those of the Andean WPV1. Since the transition/transversion ratio is sensitive to sampling error if the dataset is small [41] this dataset may be too small to estimate the Ts/Tv coefficients reliably. The same holds true for the other aVDPV datasets, the sets of iVDPV sequences, and all but one cVDPV dataset listed in Table S1: the large Nigerian cVDPV2 dataset consisting of 361 sequences considered to be originating from a single emergence of cVDPV from PV2 Sabin. It had the 2^nd^ codon position coefficients very similar to those of SVK-aVDPV2.

The observed relatively increased preference for the 2^nd^ codon position transversions in SVK-aVDPV2 evolution could be related to the previously known relatively high amino acid substitution rate in prolonged poliovirus infection [2], [3], [12], [13]. Amino acid selection might skew the Ts/Tv rates, especially those at the 2^nd^ codon position as all 2^nd^ codon position substitutions are either nonsynonymous or nonsense (stop codon) mutations. Indeed the number of vaiable amino acid sites was 2–3-fold higher in the SVK-aVDPV2 and in the Nigerian cVDPV sequence sets than in the Andean WP1 dataset. There may be several reasons for the observed differences in amino acid substitution rates between iVDPVs and WPVs, but we believe that one of them might be difference in sample collection principles. The analysed sets of wild type virus sequences typically comprise single strains from individual hosts and have been isolated from faecal samples during the early phase of infection, before most putative adaptive changes also occurring during acute infection [42] in the genome have taken place. In contrast, the current environmental VDPV isolates were likely to be derived from prolonged infection of a single person, where the generated lineages are likely to be competing with each other, and increments of improved relative fitness may be repeatedly required for the replication of a given lineage to continue. We chose to include the sets of cVDPV sequences in the analysis as the sample collection from cVDPV outbreaks are similar to those of WPV outbreaks, mostly a single strain per patient during the acute phase of paralytic disease. The 2^nd^ codon position substitution pattern during the Nigerian cVDPV outbreak was, however, not like that of WPV1. This suggests that the observed Ts/Tv differences between the WPV1 and VDPV datasets might be reflecting the amino acid substitution rates rather than prolonged replication in a given host.

### Predicted sites of codon selection

Virion surface has a central function in virus-host interactions, including binding to receptor molecules as a prerequisite for viral entry into the host cell. In addition, the capsid surface is known to include amino acid residues involved in the binding of neutralizing antibodies. Furthermore, the surface exposed inter-beta-strand loops are likely to allow amino acid substitutions more readily – without compromising viral fitness – than those parts of the capsid proteins that are involved in maintaining the beta barrel structure. Hence, during evolution, amino acid substitutions may accumulate on capsid surface both randomly and based on selection due to a variety of factors [42], [43].

Analysis of the ratio of non-synonymous versus synonymous substitutions within individual codons of the VP1 sequences of the Slovakian aVDPV2 strains revealed statistically significant evidence for positive selection at three separate regions. Only one of these regions (221–222) was located at a known neutralisation-antigenic site. However, the knowledge on the neutralization-antigenic (NAg) sites may be limited as the identification of antigenic sites based on amino acid substitutions in variants resistant to neutralising mouse monoclonal antibodies may miss important sites for various reasons [44]. On the other hand, positive selection at a surface exposed site may reflect mechanisms other than immunological selection [42], [43]. One of the amino acid sites where putative positive selection was inferred, amino acid 24, is located at the inner surface of the capsid wall, but during uncoating, the amino terminus of VP1 is translocated to the outside of capsid [45], [46], [47]. Variants with substitution at site 24 might have had a relative growth advantage under specific situations. Likewise, substitutions at amino acid positions 144 and 147, located close to the icosahedral 5-fold axis in the DE-loop of VP1, might be able to modify conformational changes in the virion shell during uncoating.

Although immunological selection sounds an unlikely mechanism in a putatively immune deficient person, we do not actually know how completely immune deficient the person shedding these viruses was, as discussed in the adjacent paper [29]. An immune-compromised person might have an immune response insufficient to clear the virus infection but capable of imposing selection pressure to a virus. Furthermore, if all the virus strains described here were indeed derived from a single excretor, selection in distinct codons would have to reflect selection pressures within single host individual, whereas during epidemics, the virus could preferentially infect immunologically naïve persons and be subjected to different selection pressures. This could be a possible explanation for the lack of positive selection among epidemic transmission of both WPVs and cVDPVs.

### Prolonged excretion but for how long?

Different approaches for molecular clock –based estimation of the date of divergence from PV2 Sabin gave variable results. Because of the known saturation of transitions in long term diversification of RNA genomes [6] we restricted the linear regression analysis of time-dependent divergence from the PV2 Sabin sequence to 2- or 4-fold degenerate sites only. The apparent rate of evolution during the 22 month window was similar to that reported by Jorba *et al*. for synonymous transversions in the Andean WPV1 evolution [6]. Accordingly, the calculated dates for separation from the PV2 Sabin sequence were somewhat longer than predicted from the simple rule of thumb, an increment of one per cent per year. This is an expected result if we accept the view that saturation will cause a decline in the overall diversification rate. Breaking down the analysis to individual designated lineage level brought about huge variation in the coefficient of the time-dependent divergence from PV2 Sabin including a negative value for one designated lineage. We believe that the sampling bias discussed above and in the adjacent paper may have contributed to the results and the calculated values for the individual lineages do not necessarily reflect the true rates of evolution. The Bayesian MCMC method was used to estimate the time of the most recent common ancestor for the SVK-aVDPV2 strains. It suggested a relatively high overall substitution rate for this diversification period. Compared to the PV2 Sabin sequence, there was a large number of nucleotide substitutions common for all the SVK-aVDPV2 strains (and therefore also for the hypothetical MRCA strain). The rate at which these mutations accumulated during the evolution of the Sabin-derived strains that existed before the appearance of the hypothetical MRCA strain cannot be inferred directly. However, if constant rates of 0.01 [6] or 0.02 (this study) substitution/site/year were assumed, the initial OPV dose would have been administered 9 to 21 years before the last strain of the episode was obtained. We conclude that it is difficult to accurately determine the date of administration of the OPV dose which resulted in the emergence of the SVK-aVDPV lineages.

### Final Remarks

The genetic analysis of VP1 sequences of a set of Slovakian aVDPV2 strains, with features similar to those of iVDPV strains, suggested that the evolutionary pattern of poliovirus during a prolonged infection may have some differences from that described for WPV1 in natural transmission. The high transition-transversion bias typical of WPV1 evolution was definitely diminished in the SVK-aVDPV2 evolution overall, and especially as regards the 2^nd^ codon position. A recently described sequence set from a large Nigerian cVDPV2 lineage showed similar Ts/Tv pattern in the 2^nd^ codon position. The latter difference may be related to the relatively more frequent amino acid acid substitutions in the VDPV sequence sets. The rate of synonymous transversions measured for the SVK-aVDPV2 during the time window of 22 months was similar to that reported to continue for at least 20 years in WPV evolution. Apart from random accumulation of mainly neutral nucleotide substitutions, typical of WPV circulation in human populations, signs of positive selection were identified at five codons in the VP1 coding sequence of the SVK-aVDPV2 strains but not among the Nigerian cVDPV2 strains. The latter difference, if confirmed by future analysis of independent iVDPV or aVDPV datasets, might reflect a continuous search for improved fitness due to competition between different virus lineages in the same host individual or different selection pressures during intra-host and person to person transmission, or both.

## Supporting Information

Figure S1 **Putative origin of the Slovakian environmental VDPV strains.** Phylogenetic analysis of VP1 sequences of studied VDPV strains and other type 2 poliovirus strains available in GenBank on 15 April, 2013. All wild PV2 strains and representatives of all known cVDPV episodes were included in the analysis. The tree was constructed using Bayesian MCMC method with GTR model of substitution and gamma distributed substitution rate variation among sites.
(PPTX)

Figure S2 **A maximum clade credibility tree constructed from SVK-aVDPV2 sequences.** Bayesian MCMC method with GTR model of substitution, gamma distributed substitution rate variation among sites and Bayesian skyline demographic model was used. Posterior probabilities are shown in each node. Colours and codes in subclusters refer to subclusters designated on the basis of the neighbour-joining tree shown in Fig. 1. in the article.
(PPTX)

Table S1 **Nucleotide substitution pattern and selected other features of VP! Coding sequences of different poliovirus strain collections.**
(DOCX)

Table S2 **Codon usage bias in four sets of poliovirus VP1 sequences.**
(DOC)
