# Supplementary figures and images for: Evolution of Type 2 Vaccine Derived Poliovirus Lineages. Evidence for Codon-Specific Positive Selection at Three Distinct Locations on Capsid Wall
Source: PLoS One. 2013 Jun 28;8(6):e66836. doi: 10.1371/journal.pone.0066836 (PMC3696017; doi:10.1371/journal.pone.0066836)

## Slide 1
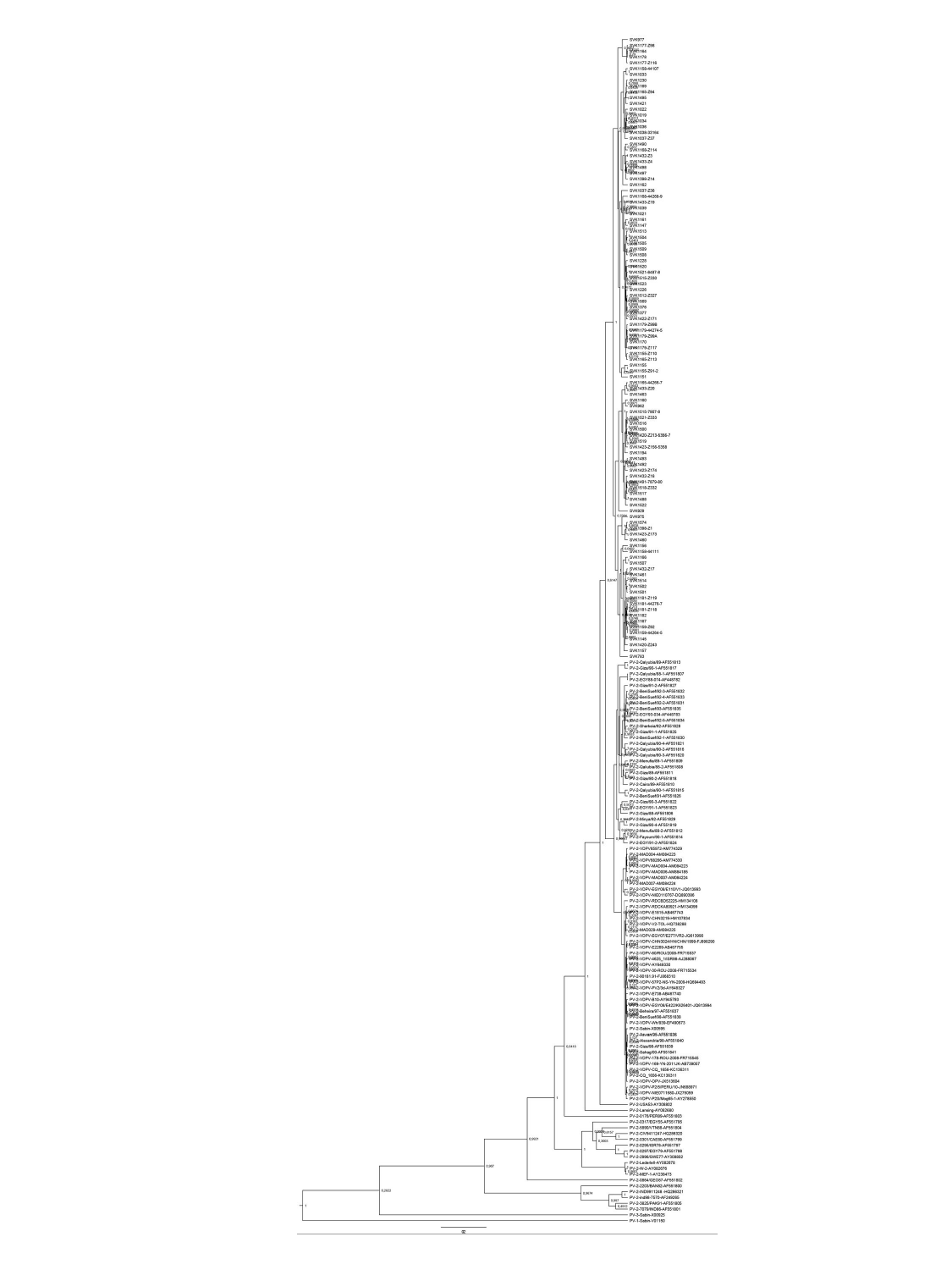

Supplement: Figure S1 — Putative origin of the Slovakian environmental VDPV strains. Phylogenetic analysis of VP1 sequences of studied VDPV strains and other type 2 poliovirus strains available in GenBank on 15 April, 2013. All wild PV2 strains and representatives of all known cVDPV episodes were included in the analysis. The tree was constructed using Bayesian MCMC method with GTR model of substitution and gamma distributed substitution rate variation among sites. (PPTX) [file pone.0066836.s001.pptx]

## Slide 1
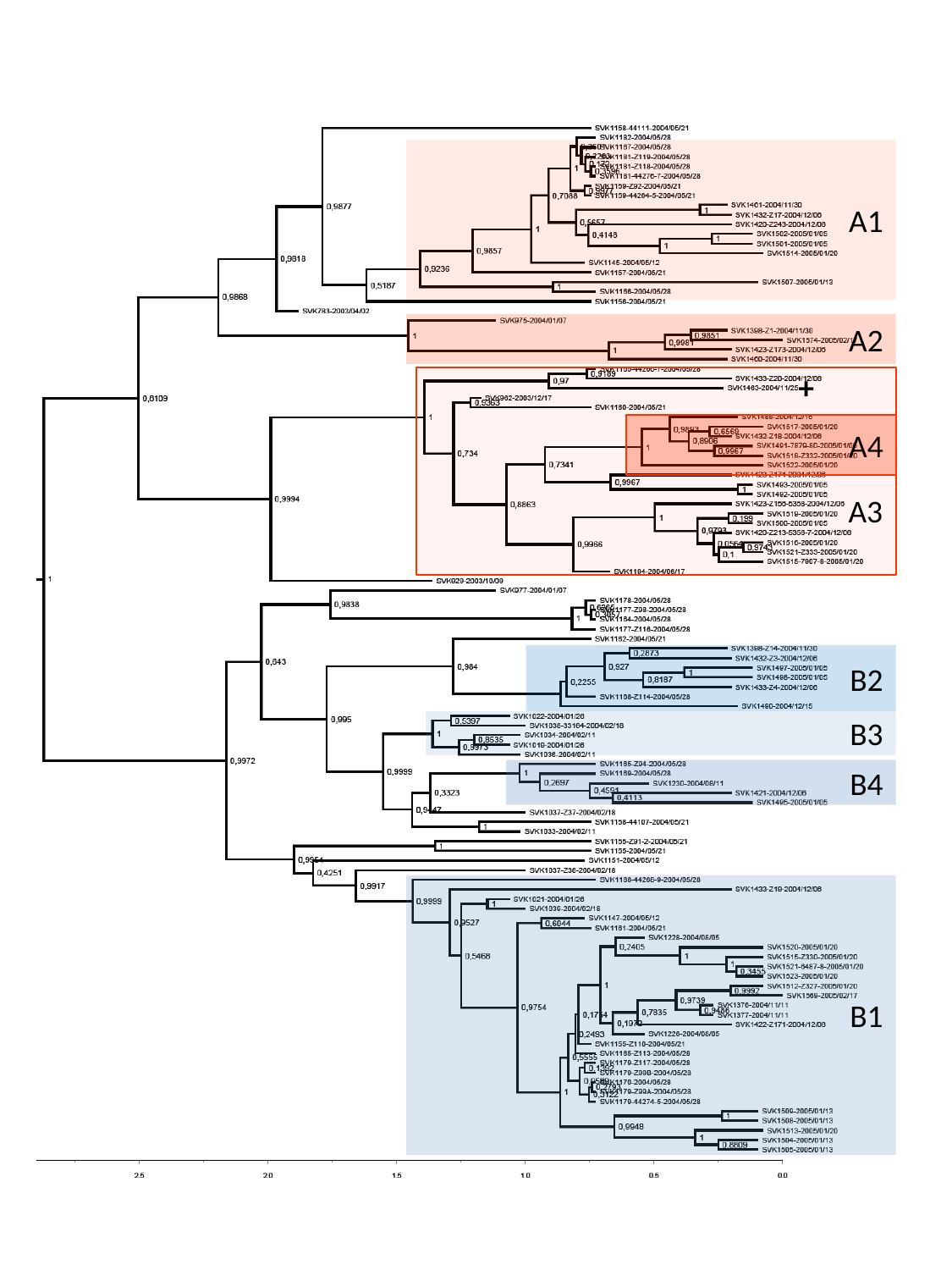

A1
A2
+
A4
A3
B2
B3
B4
B1

Supplement: Figure S2 — A maximum clade credibility tree constructed from SVK-aVDPV2 sequences. Bayesian MCMC method with GTR model of substitution, gamma distributed substitution rate variation among sites and Bayesian skyline demographic model was used. Posterior probabilities are shown in each node. Colours and codes in subclusters refer to subclusters designated on the basis of the neighbour-joining tree shown in Fig. 1. in the article. (PPTX) [file pone.0066836.s002.pptx]
