# Supplementary material for: Evolution of Type 2 Vaccine Derived Poliovirus Lineages. Evidence for Codon-Specific Positive Selection at Three Distinct Locations on Capsid Wall
Source: PLoS One. 2013 Jun 28;8(6):e66836. doi: 10.1371/journal.pone.0066836 (PMC3696017; doi:10.1371/journal.pone.0066836)
Supplement: Table S1 — Nucleotide substitution pattern and selected other features of VP! Coding sequences of different poliovirus strain collections. (DOCX) [file pone.0066836.s003.docx]

**Table S1.** Nucleotide substitution pattern and selected other features of VP1 coding sequences of different poliovirus strain collections †

| Set of sequences | *N* | First and last years of isolation | Overall mean p-distance (nt) | Number of variable nt sites (non-singletons) | Number of variable amino acid sites (non-singletons) | Relative rates of transition and transversion *(position 2)* | | |
| --- | --- | --- | --- | --- | --- | --- | --- | --- |
|  |  |  |  |  |  | Overall bias | Rate ratio purines | Rate ratio pyrimidines |
| **WPV1/Andean** | **31** | **1981 - 1991** | **0.074** | **245/906 (175)** | **34 (10)** | **10.6 *(448.5)*** | **19.1 *(709.1)*** | **25.5 *(1000)*** |
| aVDPV1/Fin* | 24 | 2008-2011 | 0.035 | 153/906 (75) | 13 (6) | 8.6 *(0.7)* | 12.3 *(0.7)* | 22.6 *(1.8)* |
| cVDPV1/Hisp# | 35 | 2000 - 2001 | 0.019 | 116/906 (59) | 15 (7) | 6.9 *(0.6)* | 10.2 *(2.1)* | 17.7 *(0.8)* |
| aVDPV2/Fin* | 37 | 2000-2011 | 0.095 | 247/903 (208) | 34 (24) | 5.0 *(3.1)* | 9.0 *(15.8)* | 11.2 *(1.2)* |
| **aVDPV2/Svk** | **102** | **2003 - 2005** | **0.055** | **350/903 (232)** | **93 (31)** | **5.6 *(2.2)*** | **9.6 *(9.4)*** | **13.0 *(1.6)*** |
| cVDPV2/Egy¤ | 30 | 1988 - 1993 | 0.067 | 268/903 (178) | 34 (11) | 11.4 *(8.7)* | 20.0 *(22.2)* | 26.1 *(14.9)* |
| cVDPV2/Nig^§^ | 361 | 2006-2011 | 0.044 | 408/903 (365) | 74 (48) | 9.31 (3.68) | 15.6 (9.60) | 22.0 (6.25) |
| WPV3/Fin | 21 | 1984 | 0.012 | 41/900 (28) | 11 (7) | 7.3 *(233.0)* | 18.3  *(1000)* | 10.9 *(220.0)* |

†Inclusion criteria: Number of strains in the collection 21 or more; WPV1 and WPV3, single lineage with a relatively recent likely ancestor, respectively; aVDPV and cVDPV sets analysed without the obvious corresponding parental Sabin strain.

N, number of sequences in the set

*Episode described in ref. [28], sequences unpublished

#Outbreak described in ref. [1], GenBank accession numbers of sequences used AF405607-AF405635, AF405666, AF405669, AF405682, AF405690, AF416341, AY046058

^¤^Outbreak described in ref. [9], GenBank accession numbers of sequences used AF551806-AF551835

^§^Outbreak described in ref. [24], GenBank accession numbers of sequences used JX274980 – JX275382 excluding the non-emergence 2005-8 strains
