# Supplementary material for: Evolution of Type 2 Vaccine Derived Poliovirus Lineages. Evidence for Codon-Specific Positive Selection at Three Distinct Locations on Capsid Wall
Source: PLoS One. 2013 Jun 28;8(6):e66836. doi: 10.1371/journal.pone.0066836 (PMC3696017; doi:10.1371/journal.pone.0066836)
Supplement: Table S2 — Codon usage bias in four sets of poliovirus VP1 sequences. (DOC) [file pone.0066836.s004.doc]

**Table S2** Codon usage bias in four sequence sets

|  |  | | **Sequence set** | | | | | | | | |
| --- | --- | --- | --- | --- | --- | --- | --- | --- | --- | --- | --- |
|  |  | | Approximate rate of codon and relative synonymous codon usage (RSCU) | | | | | | | | |
|  | |  | | **SVK-aVPV2** | | **Andean WPV1** | | **Finland WPV3** | | **Nigerian cVDPV2** | |
| Amino acid | | Codon | | Rate | RSCU | Rate | RSCU | Rate | RSCU | Rate | RSCU |
| Arginine | | CGU | | 1.2 | 0.46 | 2.1 | 0.82 | 1 | 0.42 | 0.1 | **0.03** |
|  | | CGC | | 2.9 | 1.11 | 1 | 0.39 | 2 | 0.84 | 3.9 | 1.38 |
|  | | CGA | | 3 | 1.15 | 0.8 | 0.33 | 2 | 0.84 | 3.8 | 1.33 |
|  | | CGG | | 1.9 | 0.73 | 0.2 | **0.06** | 4 | 1.69 | 1.2 | 0.44 |
|  | | AGA | | 4.1 | 1.6 | 7.9 | **3.13** | 3.7 | 1.55 | 7.7 | **2.73** |
|  | | AGG | | 2.5 | 0.94 | 3.2 | 1.26 | 1.6 | 0.66 | 0.3 | **0.1** |
| Glycine | | GGU | | 6 | 1.13 | 4.1 | 0.97 | 5 | 1.33 | 5.3 | 1 |
|  | | GGC | | 2.3 | 0.44 | 4.8 | 1.13 | 0 | **0** | 1.8 | 0.35 |
|  | | GGA | | 5.8 | 1.11 | 2.6 | 0.62 | 3 | 0.8 | 0.82 | 1.37 |
|  | | GGG | | 7 | 1.32 | 5.5 | 1.28 | 7 | 1.87 | 6.8 | 1.28 |
| Leucine | | UUA | | 2.6 | 0.91 | 2.2 | 0.7 | 3 | 1 | 1.3 | 0.45 |
|  | | UUG | | 5.9 | 2.09 | 4.1 | 1.33 | 2 | 0.67 | 5.5 | 1.94 |
|  | | CUU | | 0 | **0.01** | 0.2 | **0.06** | 3 | 1 | 0 | **0.02** |
|  | | CUC | | 3 | 1.05 | 3.4 | 1.1 | 4 | 1.33 | 2 | 0.7 |
|  | | CUA | | 2.3 | 0.82 | 4.5 | 1.45 | 2 | 0.67 | 2.3 | 0.83 |
|  | | CUG | | 3.2 | 1.12 | 4.2 | 1.36 | 4 | 1.33 | 5.8 | **2.07** |
| Proline | | CCU | | 4.7 | 0.74 | 2.1 | 0.42 | 3 | 0.5 | 5.8 | 0.88 |
|  | | CCC | | 4.7 | 0.73 | 4 | 0.79 | 6 | 1 | 5.2 | 0.79 |
|  | | CCA | | 11.7 | 1.83 | 10 | **2** | 10.6 | 1.77 | 11.2 | 1.72 |
|  | | CCG | | 4.5 | 0.7 | 3.9 | 0.79 | 4.4 | 0.73 | 4.0 | 0.61 |
| Serine | | UCU | | 1 | **0.27** | 4.4 | 0.9 | 3 | 0.82 | 1.3 | 0.35 |
|  | | UCC | | 7 | 1.82 | 10.8 | **2.24** | 5 | 1.35 | 5.7 | 1.56 |
|  | | UCA | | 8.7 | **2.26** | 7.6 | 1.57 | 8.9 | **2.4** | 7.1 | 1.95 |
|  | | UCG | | 3.2 | 0.84 | 2.9 | 0.61 | 1.1 | **0.29** | 4.8 | 1,32 |
|  | | AGU | | 1.8 | 0.47 | 2 | 0.42 | 1.1 | 0.31 | 0.3 | **0.09** |
|  | | AGC | | 1.3 | 0.33 | 1.3 | **0.27** | 3.1 | 0.83 | 2.7 | 0.74 |
| Tyrosine | | UAU | | 4.8 | 0.57 | 8.9 | 1.11 | 2.1 | **0.27** | 8 | 1 |
|  | | UAC | | 12.1 | 1.43 | 7.1 | 0.89 | 13.9 | 1.73 | 8.1 | 1 |
| Valine | | GUU | | 4.2 | 0.67 | 6.5 | 0.96 | 3.3 | 0.46 | 7.7 | 1.19 |
|  | | GUC | | 7.8 | 1.23 | 4.6 | 0.68 | 4.4 | 0.62 | 4.9 | 0.75 |
|  | | GUA | | 4.3 | 0.68 | 5.8 | 0.86 | 4.8 | 0.68 | 2.9 | 0.45 |
|  | | GUG | | 9 | 1.43 | 10 | 1.49 | 16 | **2.24** | 10.4 | 1.6 |

**Bold phase numbers,** high (2 or more) or low (0.3 or less) RSCU values
